# Oncological value of MRI in brain metastasis: exploring the potential of combining post-contrast T1 TSE (SPACE) and T1 GRE (MPRAGE) for stereotactic radiosurgery planning and surveillance

**DOI:** 10.1007/s11060-025-05365-7

**Published:** 2025-12-11

**Authors:** Emre Uysal, Philipp Reinhardt, Daniel Schmidhalter, Franca Wagner, Ekin Ermiş

**Affiliations:** 1 Department of Radiation Oncology, Inselspital, Bern University Hospital and University of Bern, Bern, Switzerland; 2 Department of Radiation Oncology, Prof. Dr. Cemil Tascioglu City Hospital, Istanbul, Turkey; 3 Division of Medical Radiation Physics, Department of Radiation Oncology, Inselspital, Bern University Hospital and University of Bern, Bern, Switzerland; 4 University Institute of Diagnostic and Interventional Neuroradiology, Inselspital, University Hospital and University of Bern, Bern, Switzerland; 5 Institute of Diagnostic and Interventional Neuroradiology, Cantonal Hospital Aarau, Aarau, Switzerland

**Keywords:** Brain metastases, Stereotactic radiosurgery, Magnetic resonance imaging, T1-SPACE, T1-MPRAGE

## Abstract

**Purpose:**

Accurate MRI-based detection of brain metastases (BM) is essential for planning stereotactic radiosurgery (SRS). Although spin-echo (SE) sequences such as T1-SPACE have shown superior lesion detectability compared with gradient-recalled echo (GRE)–based T1-MPRAGE, direct dosimetric comparisons and evaluations of clinical impact are lacking. This study aimed to quantitatively and qualitatively compare T1-SPACE and T1-MPRAGE sequences for SRS planning, focusing on lesion detectability, target volume delineation, dosimetric effects, and oncological outcomes.

**Methods:**

Quantitative, qualitative, and dosimetric analyses were performed in 51 patients who underwent MRI with T1-SPACE and T1-MPRAGE sequences prior to SRS (SPACE group). An experienced neuroradiologist identified BM on both sequences as the reference standard. For outcome evaluation, distant brain metastasis-free survival (DBMFS) and overall survival (OS) were compared between the SPACE group and a matched control group (*n* = 51) planned exclusively on the T1-MPRAGE sequence.

**Results:**

A senior resident identified significantly more BM on T1-SPACE (94.7%) than on T1-MPRAGE (82.4%). T1-SPACE also demonstrated significantly higher contrast and contrast-to-noise ratios (*p* < 0.001). Dosimetrically, T1-SPACE–based plans showed smaller planning target volumes (*p* = 0.008) and modest but significant reductions in irradiated brain volumes (V12Gy and V10Gy, both *p* < 0.05). Patients planned with T1-SPACE had longer DBMFS (10.4 vs. 5.2 months, *p* = 0.024) and better OS (*p* = 0.049) compared with the control group.

**Conclusion:**

The T1-SPACE sequence offers superior lesion detectability, more accurate target delineation, and favorable dosimetric and clinical outcomes in patients with BM. These findings support the implementation of T1-SPACE as a standard imaging sequence for SRS planning in patients with BM.

**Clinical trial number:**

Not applicable.

**Supplementary Information:**

The online version contains supplementary material available at 10.1007/s11060-025-05365-7.

## Introduction

Accurate detection of brain metastases (BM) is essential for planning stereotactic radiosurgery (SRS). Even small lesions can influence management and target volume delineation and may potentially impact oncological results. Undetected BM at baseline can lead to undertreatment, whereas inaccurate lesion delineation may result in unnecessary exposure of healthy brain tissue to radiation. As the incidence of BM continues to rise, the development and implementation of highly sensitive and reliable magnetic resonance imaging (MRI) sequences have become increasingly critical for accurate radiotherapy planning.

Historically, T1 inversion-recovery gradient-recalled echo (GRE) sequences, such as T1-weighted magnetization-prepared rapid gradient echo (MPRAGE), have been the standard isotropic 3D MRI sequence for intracranial SRS planning. More recently, superior lesion detectability with 3D turbo/fast spin-echo (SE) sequences, such as T1 sampling perfection with application-optimized contrasts using different flip angle evolution (SPACE), has been demonstrated, particularly for small BM. These advantages are based on intrinsic characteristics of 3D turboSE sequences, including: (1) Reduced sensitivity to magnetic susceptibility and B1 field inhomogeneity, resulting in more homogeneous signal across the brain and fewer artifacts; (2) Use of long echo trains with variable-flip-angle refocusing, which enhances T1 weighting while maintaining a high signal-to-noise ratio (SNR), thereby producing strong contrast between gadolinium-enhancing BM and surrounding parenchyma; (3) More uniform gray–white matter differentiation and reduced shading artifacts, facilitating the detection of subtle nodular enhancement. Few studies so far have reported improved image quality metrics, including higher SNR and contrast-to-noise ratio (CNR), with T1-SPACE [1–3]Klicken oder tippen Sie hier, um Text einzugeben. However, SE-based sequences have been shown to detect BM that may be missed on traditional GRE-based sequences, directly influencing treatment decisions [4, 5]. A recent systematic review and meta-analysis demonstrated that the overall detectability of BM was higher using SE sequences (89.2%) than with GRE sequences (81.6%), although this difference was not statistically significant [6]. Notably, using 1-mm slice thickness and focusing on small BM (< 5 mm), contrast-enhanced 3D SE sequences showed superior detectability compared to 3D GRE sequences (93.7% vs. 73.1% for 1-mm slices; 89.5% vs. 59.4% for small lesions; *p* < 0.0001).

These potential advantages in BM detectability could have further clinical implications and oncological benefits. Nevertheless, to the best of our knowledge, no study to date has conducted a direct dosimetric comparison between GRE- and SE-based sequences in this context. Retrospective analyses have suggested that the improved lesion detectability achieved with T1-SPACE sequences when used for SRS treatment planning may contribute to longer times to distant brain failure (DBF) compared with T1-MPRAGE sequences [7, 8]. While Kutuk et al. reported promising reductions in DBF, their study primarily focused on lesion detectability and overall oncological outcomes, without evaluating the underlying technical or dosimetric mechanisms. Building on these findings, the present study not only reassesses oncological endpoints but also systematically examines the dosimetric impact of T1-SPACE–based treatment planning. By integrating quantitative image-quality metrics, blinded dual-reader assessments, and intra-individual plan-to-plan comparisons, we provide new evidence to contextualize and mechanistically explore the DBF advantages suggested by Kutuk et al., clarifying whether the observed clinical benefits may result from measurable improvements in lesion conspicuity and treatment planning accuracy. More recently, prospective evidence from the CYBER-SPACE randomized phase II trial demonstrated no significant differences in overall survival (OS) or freedom from whole-brain radiotherapy (WBRT) between those planned based on T1-SPACE and T1-MPRAGE sequences [9].

Overall, while SE-based sequences may offer diagnostic advantages, their practical implications for SRS remain incompletely characterized. To address this, we quantitatively and qualitatively compared T1-SPACE and T1-MPRAGE for lesion detectability, target volume delineation, and dosimetric outcomes, alongside potential impacts on clinical endpoints.

## Materials and methods

### Methods

**Fig. 1:**
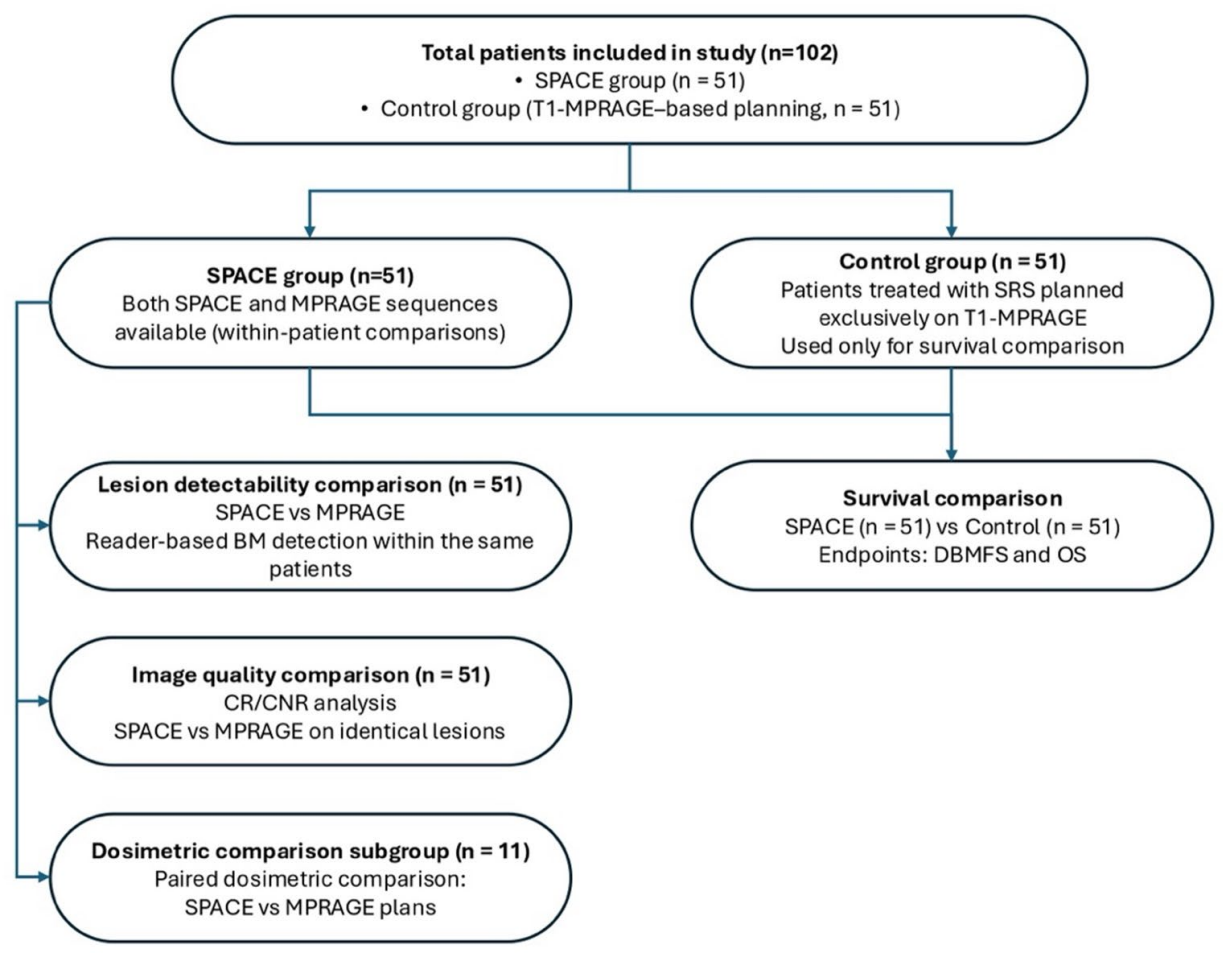
Study workflow illustrating cohort structure and analyses performed in the SPACE and control groups. Abbreviations: *BM* brain metastasis, *SRS* stereotactic radiosurgery, *OAR* organs at risk, PTV planning target volume, *DBMFS* distant brain metastasis–free survival, *OS* overall survival

This retrospective study comprised two complementary analyses of the role of T1-SPACE imaging in SRS planning for BM. In the first analysis, 51 patients who had undergone both T1-SPACE and T1- MPRAGE sequences prior to SRS were included (SPACE group). Each patient served as their own control, allowing direct intra-individual comparison of the two MRI sequences. All patients underwent standardized diagnostic MRI including pre- and post-contrast 3D T1-SPACE, 3D T1-MPRAGE, and conventional sequences (FLAIR, T2-weighted imaging, DWI, SWI). The dedicated SRS planning protocol, performed within one week before treatment, consisted of pre-contrast 3D T1-SPACE and post-contrast 3D T2-SPACE, 3D T1-MPRAGE, and coronal 2D FLAIR, following institutional standards for SRS imaging. Detailed acquisition parameters for both sequences are summarized in Supplementary Table 1. The overall cohort structure and analysis workflow are summarized in Fig. 1.

Lesion detectability, subjective image quality, target volume delineation, and associated dosimetric parameters were systematically assessed. To investigate the potential clinical impact of T1-SPACE-based planning, a second historical cohort consisting of 51 patients who underwent SRS planning based exclusively on T1-MPRAGE imaging was identified (control group). Patients in the control group were treated between May 2014 and September 2020, whereas those in the SPACE group were treated in the following period up to February 2022. Survival outcomes were then compared following T1-SPACE-based planning (*n* = 51, SPACE group) and T1-MPRAGE-only planning (*n* = 51, control group). Patients were eligible for inclusion if they were older than 18 years, had a diagnosis of one or more in-situ BM, and were treated with robotic SRS/fractionated SRS (CyberKnife^®^, Accuray Inc.) in one to five fractions. Patients were excluded if the BM had been surgically resected or biopsied, or if neuroradiological imaging was incomplete. This study was performed in line with the principles of the Declaration of Helsinki. Approval was granted by the Cantonal Ethics Committee, Bern, Switzerland (KEK BE 2022 − 00852). Informed consent was obtained from all individual participants included in the study.

### Detectability and image quality

The primary step of image evaluation focused on assessing lesion detectability. An experienced neuroradiologist determined the number of BM visible on both MPRAGE and SPACE sequences, which served as the reference standard. These results were then compared with the number of BM identified independently on each sequence by a senior radiation oncology resident.

Quantitative analysis was performed using contrast rate (CR) and CNR, calculated as follows:

- CR = [(SI_lesion_ − SI_parenchyma_)/SI_parenchyma_] × 100 [1, 3]
- CNR = (SI_lesion_ − SI_parenchyma_)/SD_parenchyma_ [1, 10]

where SI represents the mean signal intensity within the region of interest (ROI), and SD represents the standard deviation of parenchymal noise. Contrast reflects the relative signal difference between adjacent regions, whereas CNR accounts for this difference relative to image noise. Higher values indicate better lesion conspicuity and overall image quality. Lesion ROIs (mean 6.9 mm²) were manually drawn as small circular regions placed on the most homogeneously enhancing portion of each BM, avoiding necrosis or hemorrhage and without isotropic expansion of lesion contours. Reference ROIs (mean 71.6 mm²) were drawn as larger circular regions in normal-appearing white matter remote from the lesion to avoid partial-volume effects and to provide a more stable estimate of background signal and noise. All ROIs were positioned to correspond to the same anatomical locations on both T1-SPACE and T1-MPRAGE images to ensure consistency of CR and CNR measurements across sequences.

Qualitative analysis was based on visual grading by an experienced neuroradiologist and a senior radiation oncology resident blinded to clinical data but not to sequence type. Images were reviewed in random order and scored on a three-point ordinal scale (excellent, good, poor) according to lesion enhancement, separation from adjacent vessels, and susceptibility to artifacts (e.g. hemorrhage or calcifications). When the overall visual prominence of a given lesion was assessed as similar between the two sequences, the same ranking was assigned to each.

### Dosimetric comparison

Quantitative and qualitative comparisons of dosimetric parameters were performed to evaluate plan quality differences between target volumes delineated separately on T1-SPACE and T1-MPRAGE sequences within the SPACE group. Gross target volumes (GTVs) and brain organs at risk (OARs) were delineated by a senior radiation oncology resident with 4 years of clinical experience and reviewed by a senior radiation oncologist with more than 10 years of expertise. For SRS planning, the planning target volume (PTV) was defined as equivalent to the GTV without an additional margin. All treatment plans were generated in the Accuray Precision^®^ Treatment Planning System (Accuray Inc., Sunnyvale, CA, USA) using an SRS/fractionated SRS technique. Prescription doses of 18–30 Gy in 1–5 fractions were assigned according to lesion size and proximity to OARs, with identical optimization scripts applied for both T1 SPACE- and T1 MPRAGE-based plans. Fractionation schemes were 1 × 20 Gy for lesions < 10 cc, 1 × 18 Gy for lesions 10–15 cc, and 5 × 6 Gy for lesions >15 cc. For the dosimetric comparison, we identified a subgroup of patients in whom fully comparable paired plans could be generated on both T1-SPACE and T1-MPRAGE images. To ensure strict methodological consistency, these plans were reconstructed using an identical prescription (1 × 20 Gy) and the same optimization parameters. Consequently, this subgroup encompassed lesions with diverse locations, sizes, and proximities to OAR, reflecting the natural heterogeneity of the SPACE cohort rather than any feature-based selection. The prescription isodose line was set between 70 and 75% and kept consistent across plans, with a minimum accepted PTV coverage of 95%. OAR dose constraints were applied according to published guidelines [11, 12]. Plan optimization prioritized OAR sparing and maintaining Brain-PTV V12 Gy < 5 cc, whereas treatment delivery times were limited to 60 min. Dosimetric parameters including target coverage, conformity [(TV x PIV) / (TV_PIV_)^2^], heterogeneity (maximum dose / prescribed dose), gradient (PIV / PIV _HALF_) indexes and OAR doses were collected and compared for each delineation set. In these formulas, TV represents the target volume, PIV is the prescription isodose volume, TV_PIV_ is the target volume covered by the prescription isodose, and PIV_HALF_ corresponds to the volume receiving half of the prescription isodose.

### Oncological outcome evaluation

For survival analysis, two independent cohorts were evaluated. The SPACE group comprised 51 patients who underwent MRI with both T1-SPACE and T1-MPRAGE sequences prior to SRS. Treatment planning for these patients was based on target volume delineation performed on T1-SPACE. The control group consisted of 51 patients who underwent SRS planned exclusively on T1-MPRAGE sequences. The control group was assembled using predefined clinical criteria (age, primary tumor type, number of brain metastases, performance status, and treatment intent) to ensure comparability with the SPACE cohort. Patients with missing clinical information, incomplete planning MRI, or treatment indications not comparable to the SPACE group (such as salvage or repeat SRS) were excluded. Survival outcomes were compared between these two groups. Distant brain metastasis-free survival (DBMFS) was defined as the time from the first date of SRS/fractionated SRS to either the development of a new intracranial BM outside the treated target volume or death from any cause, whichever occurred first. Progression of extracranial disease was not counted as an event for DBMFS. Patients who had no event were censored at the date of last follow-up. OS was defined as the time from the first date of SRS/fractionated SRS to death from any cause, with patients alive at last follow-up censored.

### Statistics

Categorical variables were presented as numbers (percentages), while continuous variables were expressed as mean ± SD or median (interquartile range, IQR), as appropriate. The normality of continuous variables was assessed using the Kolmogorov–Smirnov and Shapiro–Wilk tests. For paired comparisons, normally distributed continuous variables were analyzed with the paired t-test, whereas non-normally distributed continuous variables and ordinal variables were compared using the Wilcoxon signed-rank test. The chi-square test or Fisher’s exact test, as appropriate, was used to compare proportions between groups. Survival analyses were performed using Kaplan–Meier analysis, calculated from the first date of SRS/fractionated SRS, and the groups were compared using the log-rank test. Prognostic factors for DBMFS were investigated with Cox regression analysis. Variables with a *p* < 0.20 in the univariate analysis, including age, sex, extracranial metastasis, the number of BM and the use of SPACE sequence, were incorporated into the multivariable Cox regression model. Statistical analyses were conducted using IBM SPSS Statistics for Windows, version 26 (IBM Corp., Armonk, N.Y., USA), with a statistical significance level set at *p* < 0.05.

## Results

### Patient characteristics

Baseline characteristics are shown in Table 1. The SPACE (*n* = 51) and control (*n* = 51) groups had comparable demographic characteristics and clinical parameters. Median age was 66 vs. 65 years (*p* = 0.941), median KPS was 90 in both groups (*p* = 0.429), and median number of BM were 2 vs. 3 (*p* = 0.921). Lung cancer was the most frequent primary tumor, accounting for 45.1% of cases in both groups. Melanoma was present in 19.6% of patients in each group, while breast cancer was observed in 13.7% of the SPACE group and 19.6% of the control group. Molecular subgroup analyses showed no significant differences in PD-L1 status (lung cancer), hormone receptor profiles (breast cancer), or BRAF mutations (melanoma) between groups (Supplementary Table 2).

**Table 1:** Patient characteristics in the SPACE and control group

	SPACE group *n* = 51 (%/IQR)	Control group* n = 51 (%/IQR)	*p*
Sex			1.000
Female	19 (37.3)	20 (39.2)	
Male	32 (62.7)	31 (60.8)	
Age in years, median	66 (59 − 71)	65 (57 − 71)	0.941
KPS, median	90 (80 − 100)	90 (80 − 100)	0.429
Number of BM, median	2 (1 − 5)	3 (1 − 5)	0.921
ECM	24 (47.1)	18 (35.3)	0.227
Primary tumor histology			0.914
Lung	23 (45.1)	23 (45.1)	
Adenocarcinoma	17 (33.3)	17 (33.3)	
Squamous	2 (3.9)	2 (3.9)	
Other	4 (7.8)	4 (7.8)	
Melanoma	10 (19.6)	10 (19.6)	
Breast	7 (13.7)	10 (19.6)	
Her2+	2 (3.9)	4 (7.8)	
Her2−	5 (9.8)	6 (11.7)	
GIS cancer	5 (9.8)	3 (5.9)	
RCC	1 (2.0)	2 (3.9)	
Other primary	5 (9.8)	5 (9.8)	
Follow-up, median	10 (7 − 15)	10 (5 − 23)	0.671

*Control group: SRS planned exclusively on T1-MPRAGE sequences

Abbreviations: *BM* brain metastases, *ECM* extracranial metastasis, *GIS* gastrointestinal system, *IQR* interquartile range, *KPS* Karnofsky Performance Status, *PTV* planning target volume, *RCC* renal cell carcinoma

### Detectability and image quality

**Fig. 2:**
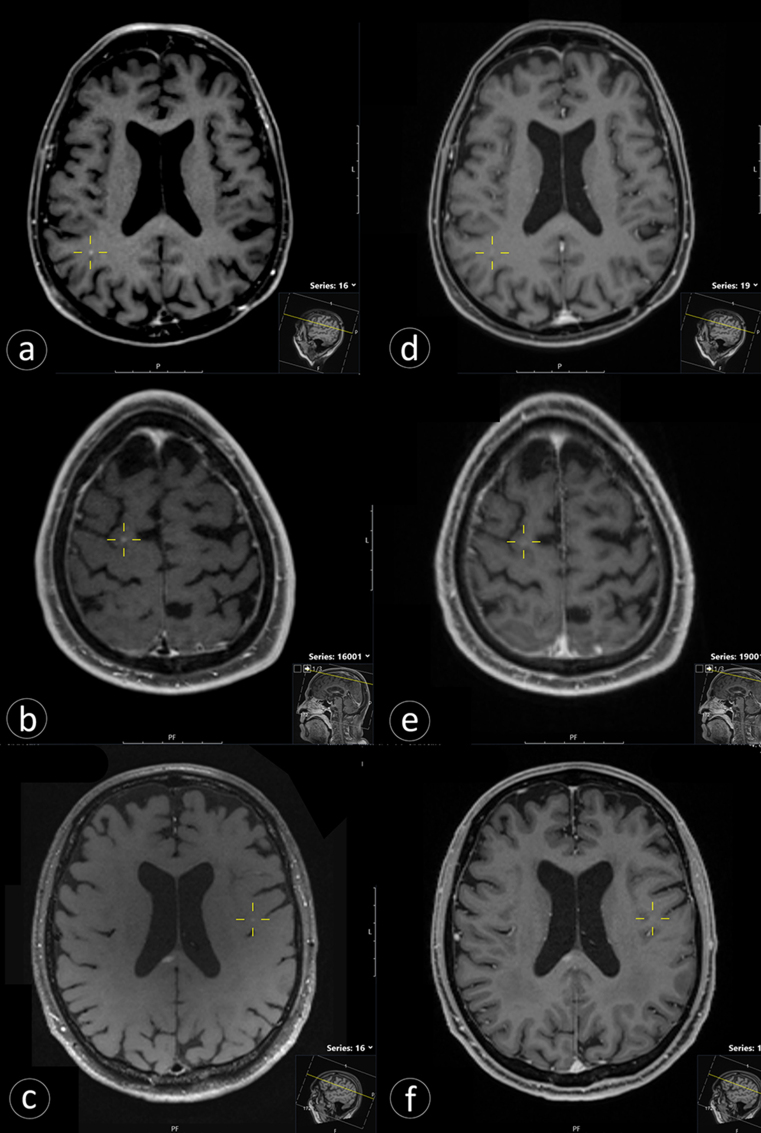
Comparison between T1-SPACE and T1-MPRAGE sequences. Images in each row are from the same patient: contrast-enhanced 3D T1-SPACE is shown on the left (**a**–**c**) and contrast-enhanced 3D T1-MPRAGE on the right (**d**–**f**). Yellow crosshairs indicate the target foci. Rows 1–2 (**a**–**b** vs. **d**–**e**): Small cortical/subcortical brain metastases are conspicuous on SPACE but not clearly visible on MPRAGE. Row 3 (**c** vs. **f**): A focus visible on SPACE has no corresponding abnormality on MPRAGE and was not confirmed as metastasis on longitudinal clinical/radiologic follow-up

An experienced neuroradiologist identified 188 BM in 51 patients using both SPACE and MPRAGE sequences (SPACE group). The senior radiation oncology resident detected 178 BM (94.7%) on T1-SPACE and 155 (82.4%) on T1-MPRAGE sequences, with 14 and 3 false-positives, respectively (Table 2; Fig. 2). Lesion CR (median 120.6 vs. 55.1) and CNR (median 30.0 vs. 20.3) were significantly higher with T1-SPACE (*p* < 0.001) (Table 2). In the visual grading analysis, the senior radiation oncology resident rated images as “excellent” in 73.5% of SPACE and 30.6% of MPRAGE scans (*p* < 0.001), whereas the experienced neuroradiologist rated image quality as “excellent” in 58.8% of SPACE and 5.9% of MPRAGE scans (*p* < 0.001) (Table 2).

**Table 2:** Comparison of quantitative and qualitative parameters between T1-SPACE and T1-MPRAGE within the SPACE group

	SPACE *n* (%) or median (IQR)	MPRAGE *n* (%) or median (IQR)	*p*
Number of correctly detected BM	178/188 (94.7%)	155/188 (82.4%)	
False positive	14	3	
CR (*n* = 49)	120.6 (64.7–158.3)	55.1 (31.4–91.5)	**< 0.001**
CNR (*n* = 49)	30.0 (19.0–40.4)	20.3 (11.9–30.7)	**< 0.001**
Visual grading – senior radiation oncology resident (*n* = 49)			**< 0.001**
– Excellent	36 (73.5%)	15 (30.6%)	
– Good	10 (20.4%)	19 (38.8%)	
– Poor	3 (6.1%)	15 (30.6%)	
Visual grading – experienced neuroradiologist (*n* = 51)			**< 0.001**
– Excellent	30 (58.8%)	3 (5.9%)	
– Good	21 (41.2%)	28 (54.9%)	
– Poor	0 (0.0)	20 (39.2%)	

Abbreviation: *BM* brain metastases, *CNR* contrast-to-noise ratio, *CR* contrast ratio

### Dosimetric analysis

For dosimetric comparison, 11 patients were replanned to receive a uniform dose of 1 × 20 Gy based on both T1 SPACE and T1 MPRAGE images. Plan comparison showed that the PTV was smaller on T1-SPACE than on T1-MPRAGE (4.57 ± 3.11 cc vs. 4.79 ± 3.13, *p* = 0.008) (Table 3). Brain V12Gy (5.72 ± 3.35 vs. 6.00 ± 3.42 cc, *p* < 0.001) and V10Gy (7.96 ± 4.65 vs. 8.30 ± 4.71 cc, *p* < 0.001) were significantly smaller with T1-SPACE, whereas the gradient index was higher (3.11 ± 0.33 vs. 3.03 ± 0.26, *p* = 0.004).

**Table 3:** Comparison of dosimetric parameters of T1-SPACE and T1-MPRAGE within the SPACE group

	SPACE mean ± SD *n* = 11	MPRAGE mean ± SD *n* = 11	*p*
PTV (cc)	4.57 ± 3.11	4.79 ± 3.13	**0.008**
Isodose line (%)	72.16 ± 0.98	71.99 ± 1.15	0.205
nCI	1.140 ± 0.051	1.155 ± 0.055	0.091
HI	1.386 ± 0.202	1.391 ± 0.226	0.052
GI	3.111 ± 0.337	3.032 ± 0.265	**0.031**
Brain-GTV V12 Gy (cc)	5.72 ± 3.35	6.00 ± 3.42	**0.010**
Brain-GTV V10 Gy (cc)	7.96 ± 4.65	8.30 ± 4.71	**0.012**

Abbreviations: *GI* gradient index, *GTV* gross tumor volume, *HI* homogeneity index, *nCI* new conformity index, *PTV* planned treatment volume

### Oncological outcomes

**Fig. 3:**
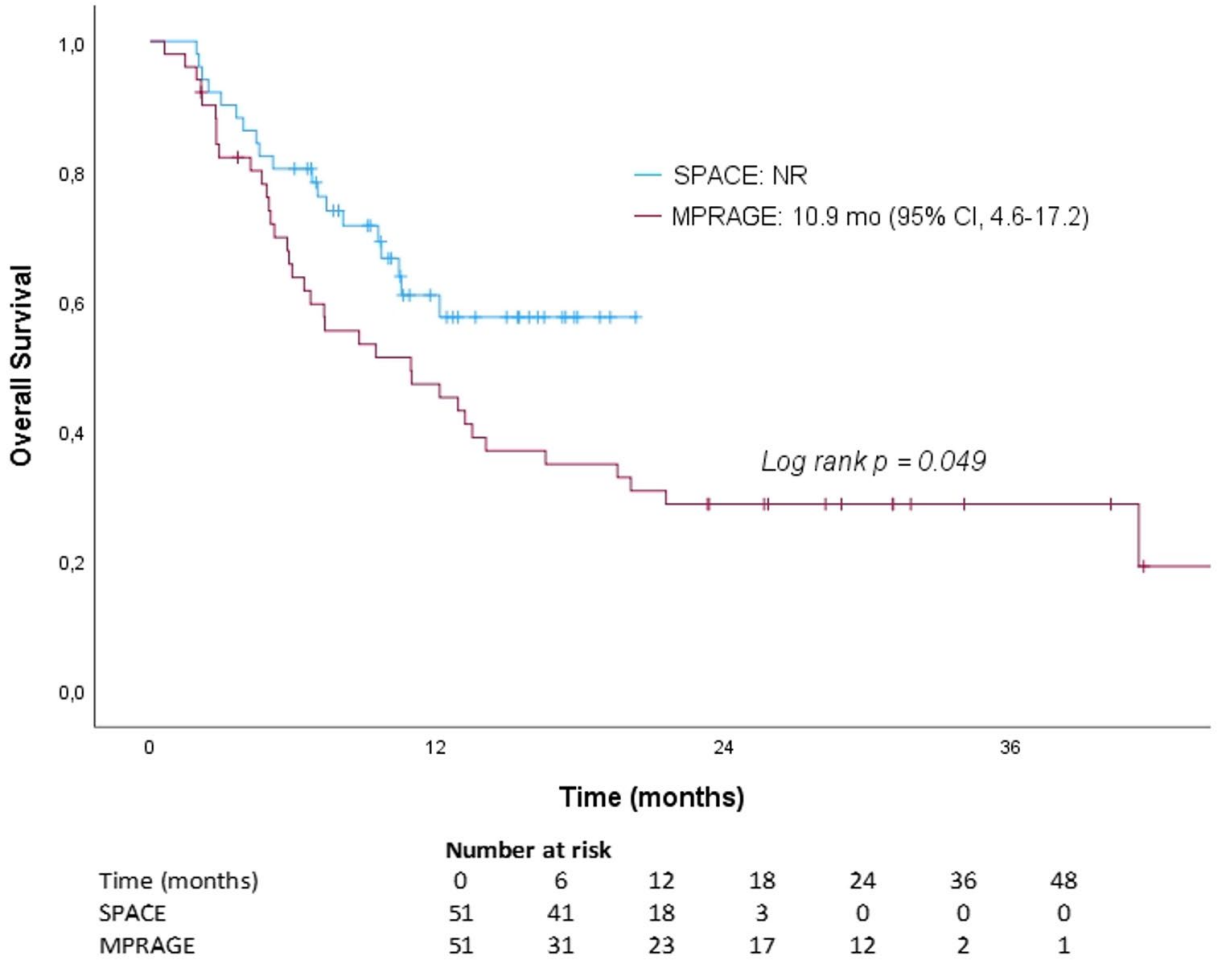
Kaplan–Meier Curve Showing Overall Survival for Patients Treated According to T1-SPACE-based Planning Versus T1-MPRAGE-only Planning. Abbreviation: *NR* not reached

The median follow-up was 10 months (range: 1–86 months) for all the patients, 10 months (range: 2–20 months) for the SPACE group and 10 months (range: 1–86 months) for the control group (*p* = 0.671). The median OS for the entire group was 13.2 months (95% CI, 8.1–18.3 months). In subgroup analysis, the median OS was 10.9 months (95% CI, 4.6–17.2 months) in the control group, whereas it was not reached in the SPACE group, showing a statistically significant difference between the two groups (*p* = 0.049). OS rates at 6 months, 1 year, and 2 years were 63.7%, 47.2%, and 28.7% in the control group, compared with 80.4%, 60.9%, and 57.5% in the SPACE group (Fig. 3).

**Fig. 4:**
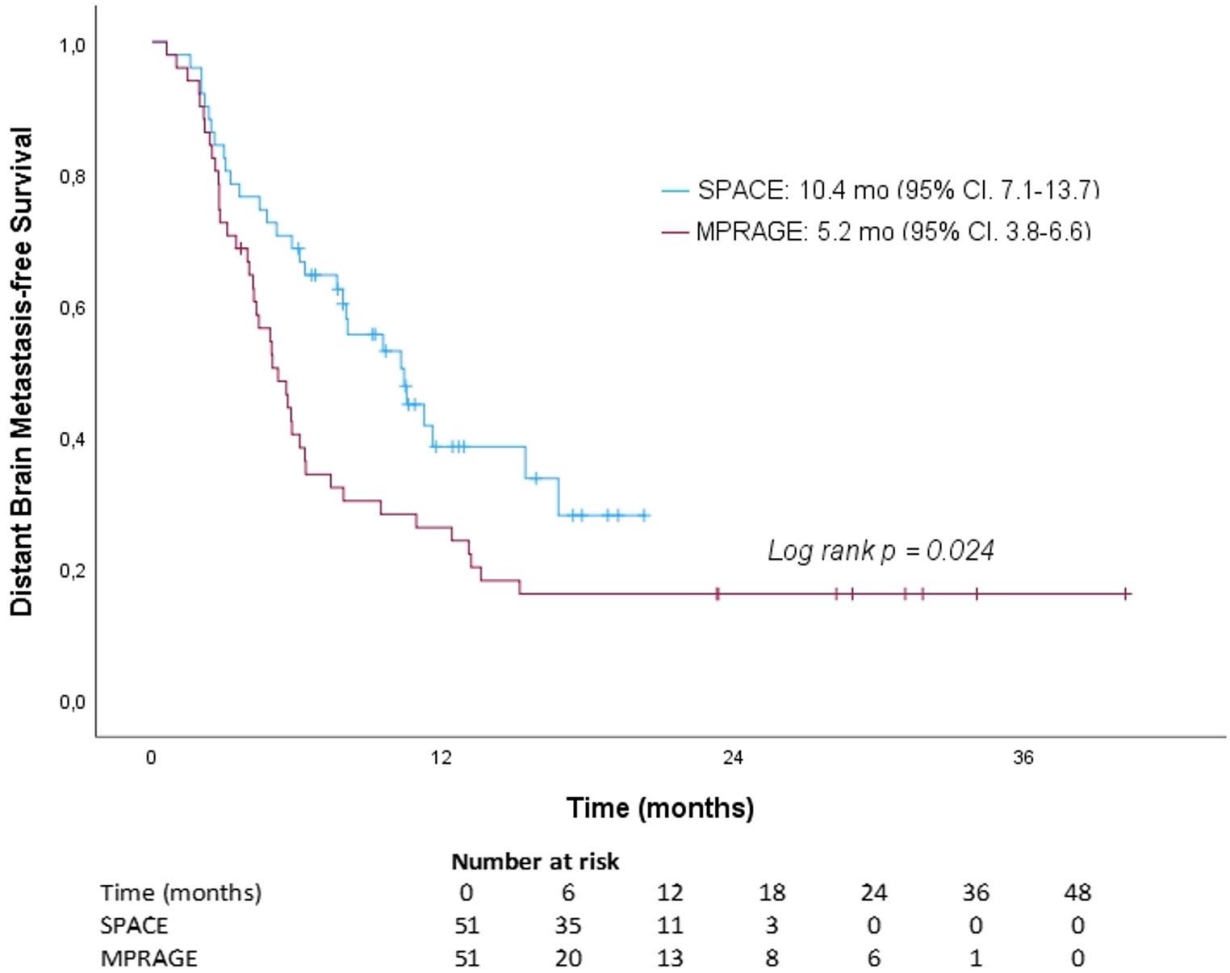
Kaplan–Meier Curve Showing Distant Brain Metastasis-Free Survival for Patients Planned with T1-SPACE Versus T1-MPRAGE-Only

The median time from SRS to DBF was 6.1 months (range: 2.5–10.2 months) in 19 patients (37.3%) in the SPACE group and 4.7 months (range: 3.0–6.6 months) in 26 patients (51.0%) in the control group. The median DBMFS was 5.2 months (95% CI, 3.8–6.6) in the control group and 10.4 months (95% CI, 7.1–13.7) in the SPACE group, showing a statistically significant improvement when planned with SPACE (*p* = 0.024). The 6-month, 1-year, and 2-year DBMFS rates were 40.4%, 26.2% and 16.1% for the control group, and 68.6%, 38.5%, and 28.1% for the SPACE group, respectively (Fig. 4).

In the univariate analysis, age, sex, primary tumor histology, performance status, and the presence of extracranial metastasis were not statistically significant predictors of DBMFS (Table 4). The difference in the number of BM showed a trend toward significance but did not reach statistical significance (*p* = 0.086). Notably, the use of the SPACE sequence was significantly associated with improved DBMFS (HR: 0.59; 95% CI: 0.37–0.94; *p* = 0.026). In multivariable analysis, age, sex, histology, performance status, and extracranial metastasis remained non-significant (Table 4). However, the number of BM was identified as an independent prognostic factor (HR 1.13; 95% CI, 1.03–1.25; *p* = 0.014). The SPACE sequence also retained a significant association with improved DBMFS (HR 0.60; 95% CI, 0.37–0.97; *p* = 0.037).

**Table 4:** Univariate and multivariable analysis for DBMFS

Characteristics	Subgroup	Univariate analysis			Multivariable analysis		
		HR	CI 95%	*p*	Hazard ratio	CI 95%	*p*
Age		1.02	0.99–1.04	0.062	1.02	0.99–1.04	0.207
Sex	Female	Ref		0.083	Ref		
	Male	1.55	0.94–2.55		1.41	0.77–2.56	0.265
Primary histology	Lung ADC	Ref		0.782			
	Lung SCC	1.56	0.54–4.53				
	Lung, others	1.19	0.49–2.93				
	Breast HER2+	0.57	0.17–1.90				
	Breast Her2−	1.13	0.51–2.52				
	Melanoma	1.02	0.51–2.04				
	GIS	1.90	0.65–5.49				
	RCC	1.38	0.56–3.40				
	Others	0.67	0.25–1.75				
BM number		1.08	0.99–1.19	0.086	1.13	1.03–1.25	**0.014**
KPS	90–100	Ref		0.885			
	80	1.14	0.65–2.00				
	≤ 70	1.11	0.59–2.11				
ECM	No	Ref		0.087	Ref		0.064
	Yes	0.66	0.41–1.06		0.63	0.38–1.03	
Use of SPACE sequence	No	Ref		Ref			
	Yes	0.59	0.37–0.94	0.026	0.60	0.37–0.97	**0.037**

Abbreviations: *ADC* adenocarcinoma, *BM* brain metastases, *CI* confidence interval, *ECM* extracranial metastasis, *GIS* gastrointestinal system, *KPS* Karnofski Performance Status, *RCC* renal cell carcinoma, *Ref* reference category, *SCC* squamous cell carcinoma

## Discussion

The prevalence of BM continues to rise, primarily due to advancements in systemic cancer treatments that have extended patient survival [13]. This trend emphasizes the critical need for precise BM detection and monitoring after treatment. Our retrospective analysis demonstrated that 3D SPACE significantly outperformed the conventional 3D MPRAGE in terms of CR and CNR and enabled the detection of a higher number of BM by less experienced MRI readers. Furthermore, when comparing treatment plans, there were small but significant differences in dosimetric parameters between the two MRI sequences. Finally, we observed a significantly prolonged time to DBMFS in patients who underwent radiosurgery for BM, as assessed using SPACE sequences.

The superior CR and CNR observed with 3D T1-SPACE directly impact lesion detectability. Higher CR and CNR allow for clearer delineation of small or subtle BM, thereby reducing the likelihood of missed lesions. The observation that SE-based sequences provide superior lesion detection compared to GRE-based sequences is not new. Nearly three decades ago, Chappell et al. demonstrated that T1-weighted SE sequences showed significantly higher contrast and CNR for small intra-axial lesions than GRE sequences [14]. These findings have subsequently been confirmed [1, 3, 4]. The detection advantage of SE-based sequences is most evident for small BM. In a systematic review, Suh et al. reported higher detection rates of BM with SE compared to GRE, although the overall difference did not reach statistical significance (*p* = 0.24) [6]. Subgroup analyses, however, revealed a significant benefit for detecting metastases < 5 mm (*p* < 0.0001). These results are consistent with reports from Zhou et al. and Park et al., who showed improved detection of small cortical and infratentorial metastases [15, 16]. Similarly, Kammer et al. reported a significantly higher detection rate of BM using a modified T1-weighted 3D TSE black-blood sequence compared to T1-MPRAGE (61 vs. 36; *p* < 0.05) [17]. Notably, the lesions detected exclusively with the TSE sequence were significantly smaller (4.3 ± 3.7 mm vs. 11.3 ± 10.7 mm; *p* < 0.01). Additional evidence suggested that SPACE sequences can better differentiate small BM from transverse vessel cross-sections due to vessel suppression [2]. Furthermore, we found a significant difference in the visual grading, favoring the SPACE sequences. These advantages have contributed to the adoption of standardized international imaging protocols for assessment of BM. Kaufmann et al. proposed an “ideal” protocol recommending the replacement of IR-GRE with 3D TSE T1-weighted imaging both before and after gadolinium administration [18]. This approach is further supported by current recommendations from the French Society of Neuroradiology and the German Taskforce on “Imaging in Stereotactic Radiotherapy” [19, 20]. In our study, a senior resident in radiation oncology identified significantly more BM using T1-SPACE sequences, implying an advantage for less experienced MRI readers. Despite this, the number of false-positive BM detected was higher, as also observed by Nagao et al. [21]. Thus, the main limitation of post-contrast T1 SPACE sequences in detecting BM is the risk of false-positive findings. This issue arises primarily from incomplete blood signal suppression and the sequence’s high sensitivity to small enhancing foci, which can lead to misidentification of vascular structures, microvascular enhancement, or inflammatory lesions as metastases [22, 23]. To minimize this risk, an additional post-contrast T1-weighted sequence without blood suppression is often included alongside T1-SPACE. This approach safeguards against overestimating the metastatic burden. Alternatively, applying stronger blood suppression techniques, such as DANTE pulses, may further reduce false-positives by improving blood signal suppression and enhancing lesion-to-parenchyma contrast [23, 24]. Using 3T MRI instead of 1.5T can improve overall image quality, CNR, and lesion detectability. However, this advantage does not necessarily translate into fewer false positives, as previous studies have shown that lesion detectability and conspicuity at 1.5T are comparable to those at 3T, with both field strengths yielding similar detection rates of BM [25]. To mitigate these known limitations, our institution adopted a combined T1-MPRAGE and T1-SPACE protocol for SRS planning beginning in January 2020. In addition, the DANTE sequence was integrated into our standard diagnostic workflow for BM to further enhance overall imaging quality.]. Although diagnostic accuracy is critical in cancer patients with BM, the clinical relevance of T1-SPACE ultimately depends on its impact on treatment planning and dose distribution. Enhanced visualization of lesion margins could directly affect target volume definition, conformity, and sparing of normal tissue. Given that lesion delineation plays a decisive role in determining target volumes and the radiation dose delivered to surrounding brain tissue, it is essential to assess whether the use of SPACE modifies dosimetric parameters in a manner that could affect toxicity or overall feasibility. We conducted a detailed dosimetric comparison of T1-SPACE- and T1-MPRAGE-based treatment plans, focusing on target coverage and OAR. This comparison was intentionally performed within the same patients to isolate the effect of contouring differences while eliminating inter-patient variability and ensuring that all paired plans were generated under identical prescription and optimization conditions. Our results revealed a subtle but statistically significant difference between T1-SPACE- and T1-MPRAGE-based plans. The PTV was slightly smaller when delineated on T1-SPACE (4.57 ± 3.11 cc) than on T1-MPRAGE (4.79 ± 3.13 cc, *p* = 0.008). This reduction aligns with previous reports suggesting that the superior conspicuity and sharper contrast of T1-SPACE sequences enable more precise definition of tumor margins, thereby limiting unnecessary inclusion of adjacent brain tissue [1, 7]. Importantly, smaller PTVs did not compromise plan quality, as conformity index, homogeneity index, and isodose line coverage were comparable between T1-SPACE- and T1-MPRAGE-based delineation. T1-SPACE-based delineation also resulted in improved sparing of normal brain tissue. Both Brain–GTV V12 Gy (5.72 vs. 6.00 cc; *p* = 0.010) and Brain–GTV V10 Gy (7.96 vs. 8.30 cc; *p* = 0.012) were significantly reduced. These reductions could be clinically meaningful, as the healthy brain volume irradiated up to 10–12 Gy is a well-established predictor of radiation necrosis and other late toxicities [26, 27]. Although the absolute differences in V12Gy and V10Gy were small, emerging evidence indicates that even low-dose exposure to normal brain (including the 5–10 Gy range) contributes incrementally to the risk of radiation necrosis, supporting the potential relevance of these modest reductions in irradiated volume during SRS planning (Ref XX) [28]. However, Mangesius et al. reported opposing results, showing that lesion volumes delineated on T1-SPACE were on average 20% larger than those on T1-MPRAGE [29]. The authors attributed these larger volumes to sequence-dependent differences in lesion conspicuity and boundary definition, noting that SPACE produces a slightly wider apparent lesion margin (approximately 0.3 mm) compared with MPRAGE. Because even small boundary expansions translate into disproportionately large volumetric changes for small BM, the effect was most pronounced for lesions < 1 cc, with volume differences reaching up to + 54%, and became minimal for lesions >1 cc. Importantly, the mean lesion volume in their cohort was only 0.56 cc, compared with 4.5 cc in our study, suggesting that lesion size likely plays a major role in the observed discrepancies. The study also demonstrated that post-contrast timing further contributes to apparent lesion growth across all sequences, although these timing effects alone could not explain the consistently larger volume measurements obtained with SPACE. Collectively, these findings indicate that the larger SPACE-derived volumes primarily reflect technical sequence characteristics and their amplified impact on very small BM. Taken together, our results indicate that T1-SPACE can refine target delineation, reduce PTV size, and spare normal brain tissue without compromising plan quality. They also emphasize the need for standardized imaging protocols and consistent contrast timing when comparing volumetric delineations across MRI sequences for SRS planning.

Whereas the dosimetric gains underscore the technical strengths of T1-SPACE-based imaging, their true relevance lies in whether these translate into improved clinical outcomes. In the overall cohort, the median OS was 13.2 months, aligning with previously reported post-SRS outcomes, which reported OS of 8–13 months [9, 30]., Patients in the SPACE group demonstrated a small but significant survival advantage, with the median OS not being reached. These findings are consistent with those reported by Kutuk et al., who similarly observed prolonged OS in patients planned with an T1-MPRAGE + T1-SPACE approach compared to a historical cohort planned with T1-MPRAGE alone (*p* < 0.001) [7]. However, given the retrospective, non-randomized design of our study, these observations are susceptible to unmeasured confounders despite balanced baseline characteristics and should be interpreted with caution. In contrast, the prospective phase II CYBER-SPACE trial showed a numerical OS advantage of T1-MPRAGE-based over T1-SPACE-based treatment planning, although the difference did not reach statistical significance [9](*p* = 0.585) [9]. These neutral results of the CYBER-SPACE trial, representing the highest level of current evidence, indicate that any observed survival differences associated with enhanced lesion detection should be regarded primarily as hypothesis-generating. Nonetheless, precise lesion detection remains critical in SRS, where BM number, size, and location dictate eligibility, dose, and fractionation. Despite the benefits of SRS in terms of local control and reduced toxicity compared to WBRT [30–32], DBF remains a major clinical issue, occurring in up to 50% of patients within six months of treatment [33]. A retrospective cohort study by Kutuk et al. demonstrated a significantly prolonged median time to DBF with the incorporating post-contrast 3D-T1 SPACE into T1-MPRAGE-based SRS planning (13.5 vs. 5.1 months overall *p* = 0.004; 18.4 vs. 6.3 months at first SRS *p* = 0.001) and a 60% reduction in DBF when T1-SPACE is used in addition to T1-MPRAGE (HR: 0.40; 95% CI: 0.28–0.57, *p* < 0.001) [7]. Similarly, Akdemir et al. found a significantly longer time to DBF following dual-sequence (T1-MPRAGE plus 3D-TSE) planning compared with T1-MPRAGE alone (11.4 vs. 6.8 months, *p* = 0.029) [8]. More recently the CYBER-SPACE trial randomized 202 patients undergoing SRS for untreated BM to lesion delineation with either SPACE or MPRAGE. The study found no significant difference in the primary endpoint of freedom from WBRT indication (HR = 0.84, 95% CI: 0.43–1.63, *p* = 0.590) [9]. Despite the negative primary endpoint, the authors concluded that close post-SRS monitoring and immediate re-treatment for new lesions contributed to a reduced need for WBRT and lower rates of neurological death. Collectively, these findings highlight that the clinical impact of T1-SPACE based treatment planning on OS and DBF remains hypothesis-generating and requires prospective validation.

Beyond survival outcomes, the clinical relevance of T1-SPACE lies in its potential to improve patient management and quality of life through more accurate lesion detection and follow-up imaging. Precise identification of BM is crucial for both oncological outcomes and quality of life, particularly as treatment strategies have increasingly shifted from WBRT to focal approaches such as SRS [30, 34]. Our findings indicate that T1-SPACE enhances lesion detectability, especially for small BM. Its direct oncological impact has yet to be clarified and prospective studies with long-term follow-up are needed to better define its role. Nevertheless, integrating T1-SPACE into surveillance protocols may facilitate earlier detection of recurrent or new lesions, potentially postponing the need for WBRT and helping to preserve neurocognitive function, as suggested by El Shafie et al. [9]. Importantly, detecting additional sub-centimeter metastases does not necessarily warrant switching from SRS to WBRT. In many contemporary practices, very small lesions may be managed with close MRI surveillance rather than immediate intervention, particularly when a CNS-active systemic therapy is already in place. This approach may avoid overtreatment while allowing early intervention if such lesions demonstrate continuous growth. Moreover, because T1-SPACE offers improved lesion conspicuity and measurement reproducibility, longitudinal follow-up is likely to be more reliable compared with traditional sequences. In this context, the use of a sensitive sequence such as T1-SPACE for both planning and follow-up offers an important advantage. Applying the same high-resolution, low-artifact imaging protocol across serial examinations not only supports earlier detection of new or evolving lesions but also strengthens the basis for individualized surveillance strategies that incorporate lesion count, total intracranial tumor burden, and patient-specific systemic therapy considerations. Taken together, the integration of T1-SPACE into surveillance workflows may contribute to more precise longitudinal monitoring and better-informed clinical decision-making throughout the course of metastatic disease management.

Beyond its clinical application, the superior image quality of T1-SPACE offers a strong basis for the development of artificial intelligence (AI) tools for automated detection and delineation. The superior image quality of T1-SPACE provides an excellent foundation for improving reproducibility, reducing inter-observer variability, and streamlining treatment planning. Recent evidence supports this concept: a deep-learning study demonstrated that high-quality annotations derived from T1-SPACE significantly improved AI performance in detecting and segmenting BM, even when applied to standard T1-MPRAGE sequences [35]. This approach leverages the strengths of T1-SPACE without requiring its routine use in every clinical workflow, thereby making AI solutions more accessible and resource-efficient. In the future, the strength of AI may lie in achieving high-quality, standardized delineation during treatment planning and enhancing lesion detectability in follow-up imaging.

This study has some limitations. First, its retrospective, single-center design and the use of separate patient cohorts for survival analysis introduce a potential risk of selection bias, even though baseline characteristics were comparable between groups. Specifically, the control group was drawn from a historical period prior to the implementation of T1-SPACE imaging, whereas the SPACE group underwent both T1-SPACE and T1-MPRAGE sequences. MRI examinations were performed using 1.5T and 3T scanners, introducing variability in image resolution and contrast characteristics that could influence lesion detection and volumetric assessment. In addition, the non-contemporaneous nature of the cohorts raises the possibility of temporal or protocol evolution biases that may have affected survival outcomes. Potential confounders (e.g. concomitant systemic therapy, lesion burden, and follow-up duration) may also have differed between groups. Second, the relatively small sample size (*n* = 51) limits the statistical power and generalizability of the findings. Furthermore, no external validation cohort was included, and inter-observer variability in lesion delineation could not be fully excluded. These factors should be considered when interpreting the results, and future prospective multicenter studies are warranted to confirm and expand upon these findings.

## Conclusion

This study demonstrated that T1-weighted 3D SPACE imaging provides superior image quality and enhanced detectability of BM. These advantages translated into measurable improvements in treatment planning, as T1-SPACE-based delineation resulted in smaller PTVs and greater brain sparing without compromising dosimetric quality. Notably, patients planned with T1-SPACE exhibited significantly prolonged DBMFS, suggesting a potential clinical benefit extending beyond imaging performance alone.

Although these findings underscore the technical and dosimetric strengths of T1-SPACE sequences, validation of their broader oncological relevance is warranted in larger, prospective, multicenter studies employing standardized imaging and treatment protocols. Incorporating T1-SPACE into SRS planning may enhance diagnostic confidence, enable more precise target delineation, and ultimately help optimize the therapeutic ratio in the management of BM.

## Supplementary Information

Below is the link to the electronic supplementary material.

Supplementary Material 1

## Data Availability

The datasets generated during and/or analysed during the current study are available from the corresponding author on reasonable request.

## Abbreviations

BM: Brain metastases
CNR: Contrast-to-noise ratio
CR: Contrast rate
DBF: Distant brain failure
DBMFS: Distant brain metastasis-free survival
GRE: Gradient-recalled echo
GTV: Gross target volume
IQR: Interquartile range
MRI: Magnetic resonance imaging
MPRAGE: Magnetization-prepared rapid gradient echo
OAR: Organ at risk
OS: Overall survival
PTV: Planning target volume
ROI: Region of interest
SD: Standard deviation
SE: Spin-echo
SI: Signal intensity
SNR: Signal-to-noise ratio
SPACE: Sampling perfection with application-optimized contrasts using different flip angle evolutions
SRS: Stereotactic radiosurgery
WBRT: Whole-brain radiotherapy
