# Supplementary material for: Oncological value of MRI in brain metastasis: exploring the potential of combining post-contrast T1 TSE (SPACE) and T1 GRE (MPRAGE) for stereotactic radiosurgery planning and surveillance
Source: J Neurooncol. 2025 Dec 11;176(1):117. doi: 10.1007/s11060-025-05365-7 (PMC12698826; doi:10.1007/s11060-025-05365-7)
Supplement: Supplementary file 1 — Supplementary Material 1 [file 11060_2025_5365_MOESM1_ESM.docx]

**Supplementary Material**

**Supplementary Table 1:** MRI acquisition parameters for T1-SPACE and T1-MPRAGE sequences at 1.5T and 3T.

|  | SPACE | | MPRAGE | |
| --- | --- | --- | --- | --- |
|  | 1.5T | 3T | 1.5 | 3T |
| Acqusition matrix | 256 x 256 | 256 x 205 | 256 x 256 | 256 x 256 |
| Flip angle (°) | 120 | 120 | 8 | 8 |
| Slice thickness (mm) | 1 | 1 | 1 | 1 |
| TR (milisecond) | 750 | 600 | 2030 | 2300 |
| TE (milisecond) | 9.5 | 7.6 | 2.45 | 2.26 |
| Number of averages | 1 | 2 | 1 | 1 |
| Inversion time (milisecond) | N/A | N/A | 1100 | 900 |
| TA (minute) | 6:35 | 3:47 | 4:41 | 4:42 |

**Supplementary Table 2:** Subgroup molecular analyses

|  | **SPACE group** | **Control group** | **p** |
| --- | --- | --- | --- |
|  | Lung Cancer  n=23 (%) | Lung Cancer  n=23 (%) |  |
| PDL-1 |  |  | 0.372 |
| <1 | 12 (63.2) | 10 (58.8) |  |
| 1-49 | 6 (31.5) | 3 (17.8) |  |
| ≥50 | 1 | 4 |  |
| Unknown | 4 | 6 |  |
|  | Breast Cancer  n= 7 (%) | Breast Cancer  n= 10 (%) |  |
| Hormone receptor profile |  |  | 0.820 |
| Basal like | 2 (28.6) | 3 (30.0) |  |
| Luminal A | 3 (42.9) | 2 (20.0) |  |
| Her2 / Luminal B | 2 (28.6) | 5 (50.0) |  |
|  | Melanoma  n= 10 (%) | Melanoma  n= 8 (%) |  |
| BRAF mutation |  |  | 0.342 |
| BRAF mutated | 4 (40.0) | 3 (37.5) |  |
| BRAF wild-type | 6 (60.0) | 3 (37.5) |  |
| Unknown | 0 (0.0) | 2 (25.0) |  |
